# 2,2′-(Disulfanedi­yl)dibenzoic acid–*N*,*N*′-bis­(4-pyridyl­meth­yl)ethane­dithio­amide (1/1)

**DOI:** 10.1107/S1600536810036755

**Published:** 2010-09-18

**Authors:** Hadi D. Arman, Tyler Miller, Pavel Poplaukhin, Edward R. T. Tiekink

**Affiliations:** a Department of Chemistry, The University of Texas at San Antonio, One UTSA Circle, San Antonio, Texas 78249-0698, USA; b Chemical Abstracts Service, 2540 Olentangy River Rd, Columbus, Ohio 43202, USA; c Department of Chemistry, University of Malaya, 50603 Kuala Lumpur, Malaysia

## Abstract

The asymmetric unit of the title co-crystal, C_14_H_14_N_4_S_2_·C_14_H_10_O_4_S_2_, comprises a twisted 2,2′-(disulfanedi­yl)dibenzoic acid mol­ecule [dihedral angle between the benzene rings = 83.53 (14)°] and a U-shaped mol­ecule of *N*,*N*′-bis­(4-pyridyl­meth­yl)ethane­dithio­amide in which intra­molecular N—H⋯S hydrogen bonds are observed. Two mol­ecules of each form a centrosymmetric ring, with an extended chair conformation, mediated by carbox­yl–pyridine O—H⋯N hydrogen bonds between the carboxylic acid groups of two 2,2′-(disulfanediyl)dibenzoic acid molecules and pyridine-N atoms of two *N*,*N*’-bis(4-pyridylmethyl)ethanedithioamide molecules. The tetra­meric aggregates are linked into a supra­molecular chain along the *b* axis *via* amide–carbonyl N—H⋯O hydrogen bonds.

## Related literature

For related studies on co-crystal formation involving 2-[(2-carb­oxy­phen­yl)disulfan­yl]benzoic acid, see: Broker & Tiekink (2007 ▶, 2010 ▶); Broker *et al.* (2008 ▶); Arman *et al.* (2010 ▶).
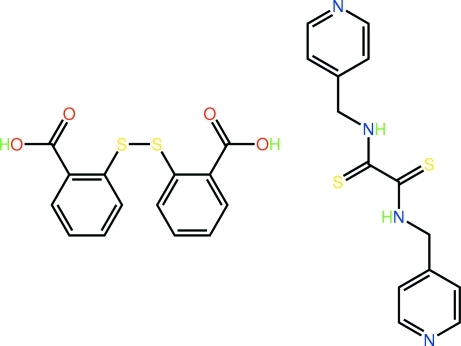


## Experimental

### 

#### Crystal data

C_14_H_14_N_4_S_2_·C_14_H_10_O_4_S_2_
*M* *_r_* = 608.75
Monoclinic, 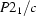

*a* = 18.502 (5) Å
*b* = 10.624 (3) Å
*c* = 15.026 (4) Å
β = 110.235 (5)°
*V* = 2771.3 (13) Å^3^
*Z* = 4
Mo *K*α radiation
μ = 0.39 mm^−1^
*T* = 98 K
0.38 × 0.26 × 0.10 mm

#### Data collection

Rigaku AFC12/SATURN724 diffractometer
16758 measured reflections
6348 independent reflections
5349 reflections with *I* > 2σ(*I*)
*R*_int_ = 0.050

#### Refinement

*R*[*F* ^2^ > 2σ(*F* ^2^)] = 0.063
*wR*(*F* ^2^) = 0.156
*S* = 1.13
6348 reflections
373 parameters
4 restraints
H atoms treated by a mixture of independent and constrained refinement
Δρ_max_ = 0.40 e Å^−3^
Δρ_min_ = −0.30 e Å^−3^

### 

Data collection: *CrystalClear* (Molecular Structure Corporation & Rigaku, 2005 ▶); cell refinement: *CrystalClear*; data reduction: *CrystalClear*; program(s) used to solve structure: *SHELXS97* (Sheldrick, 2008 ▶); program(s) used to refine structure: *SHELXL97* (Sheldrick, 2008 ▶); molecular graphics: *ORTEPII* (Johnson, 1976 ▶) and *DIAMOND* (Brandenburg, 2006 ▶); software used to prepare material for publication: *publCIF* (Westrip, 2010 ▶).

## Supplementary Material

Crystal structure: contains datablocks global, I. DOI: 10.1107/S1600536810036755/hg2714sup1.cif

Structure factors: contains datablocks I. DOI: 10.1107/S1600536810036755/hg2714Isup2.hkl

Additional supplementary materials:  crystallographic information; 3D view; checkCIF report

## Figures and Tables

**Table 1:** Hydrogen-bond geometry (Å, °)

*D*—H⋯*A*	*D*—H	H⋯*A*	*D*⋯*A*	*D*—H⋯*A*
O2—H1*o*⋯N4^i^	0.84 (3)	1.73 (3)	2.565 (4)	168 (4)
O4—H2*o*⋯N1^ii^	0.85 (3)	1.76 (3)	2.529 (3)	151 (4)
N2—H1*n*⋯S2	0.88 (2)	2.37 (2)	2.930 (3)	121 (2)
N3—H2*n*⋯O3^iii^	0.88 (3)	2.58 (3)	3.312 (4)	142 (2)
N3—H2*n*⋯S1	0.88 (3)	2.45 (3)	2.959 (3)	117 (2)

Symmetry codes: (i) 
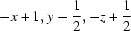
; (ii) 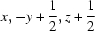; (iii) 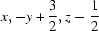.

**Fig. 1.**
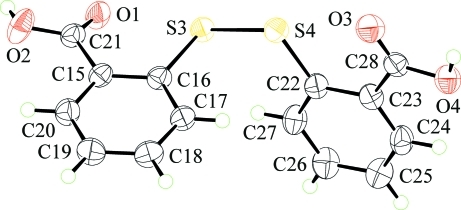
Molecular structure of 2-[(2-carboxyphenyl)disulfanyl]benzoic acid found in the co-crystal of (I) showing atom-labelling scheme and displacement ellipsoids at the 50% probability level.

**Fig. 2.**
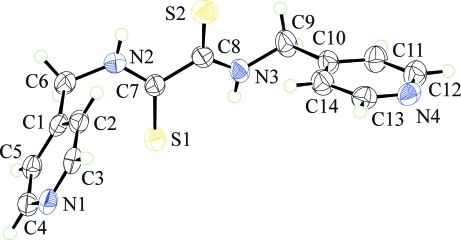
Molecular structure of N,N'-bis(4-pyridylmethyl)ethanedithioamide found in the co-crystal of (I) showing atom-labelling scheme and displacement ellipsoids at the 50% probability level.

**Fig. 3.**
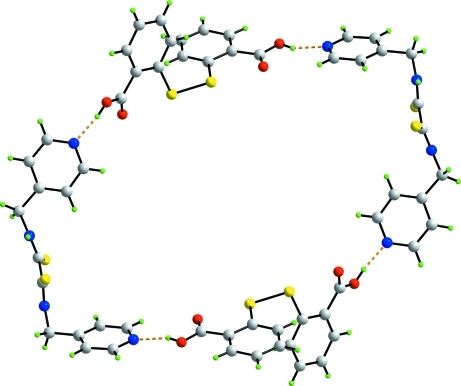
Supramolecular ring in (I). The O—H···N hydrogen bonds are shown as orange dashed lines.

**Fig. 4.**
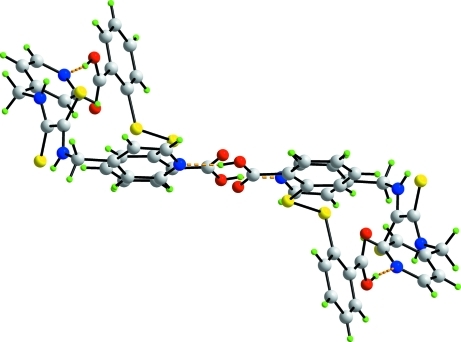
Supramolecular ring in (I) viewed side-on to emphasize the chair-like conformation. The O—H···N hydrogen bonds are shown as orange dashed lines.

**Fig. 5.**
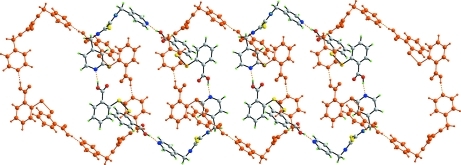
View of the supramolecular chain along the b axis in (I). The O—H···N hydrogen bonds are shown as orange dashed lines. Every second tetrameric aggregate is shown in orange. The N—H···O hydrogen bonds are obscured in this view.

**Fig. 6.**
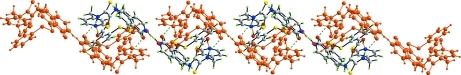
Side-on view of the supramolecular chain along the b axis in (I). The O—H···N and N—H···O hydrogen bonds are shown as orange and green dashed lines, respectively. Every second tetrameric aggregate is shown in orange.

## supplementary crystallographic information

### Comment

Co-crystallization of 2-[(2-carboxyphenyl)disulfanyl]benzoic acid with various pyridine donors has led to the isolation of a variety of supramolecular motifs (Broker & Tiekink, 2007; Broker *et al.*, 2008; Broker & Tiekink, 2010; Arman *et al.*, 2010). Herein, the structure determination of the 1:1 co-crystal obtained from the co-crystallization of 2,2'-(disulfanediyl)dibenzoic acid with *N*,*N*'-bis(4-pyridylmethyl)ethanedithioamide is described. The asymmetric unit of the resulting 1:1 co-crystal contains one molecule of 2,2'-(disulfanediyl)dibenzoic acid, Fig. 1, and *N*,*N*'-bis(4-pyridylmethyl)ethanedithioamide, Fig. 2.

In the acid, the expected conformation is observed (Broker & Tiekink, 2007), stabilized in part by two close *S*···*O*(carbonyl) interactions, *i.e*. S3···O1 = 2.759 (2) Å and S4···O3 = 2.687 (3) Å; the dihedral angle formed between the benzene rings = 83.53 (14) °. The molecule of *N*,*N*'-bis(4-pyridylmethyl)ethanedithioamide adopts a U-shaped conformation as both pyridyl groups lie to the same side of the molecule with the C2—C1—C6—N2 and N3—C9—C10—C11 torsion angles being 40.8 (4) and 146.4 (3) °, respectively. Intramolecular N—H···S hydrogen bonds are noted, Table 1.

The components of the co-crystal are linked into a supramolecular ring whereby each carboxylic acid group of two 2,2'-(disulfanediyl)dibenzoic acid molecules form an O—H···N hydrogen bond with a pyridine-N of two *N*,*N*'-bis(4-pyridylmethyl)ethanedithioamide molecules, Fig. 3 and Table 1. The ring has a extended chair conformation as seen from the view in Fig. 4. Chairs stack to form a supramolecular chain with the main connections between the tetrameric aggregates being amide-N3—*H*···O3-carbonyl hydrogen bonds, Figs 5 & 6 and Table 1.

### Experimental

Equimolar amounts of 2-[(2-carboxyphenyl)disulfanyl]benzoic acid (Fluka) and *N*,*N*'-bis(4-pyridylmethyl)ethanedithioamide were dissolved in an 1:1 ethanol/chloroform mixture. Crystals were harvested after a few days of slow evaporation.

### Refinement

C-bound H-atoms were placed in calculated positions (C–H 0.95–0.99 Å) and were included in the refinement in the riding model approximation with *U*_iso_(H) set to 1.2*U*_eq_(C). The O– and N-bound H-atoms were located in a difference Fourier map and were refined with distance restraints of O–H 0.84±0.01 Å and N—H = 0.88±0.01 Å, and with *U*_iso_(H) = *yU*_eq_(carrier atom); *y* = 1.5 for O and *y* = 1.2 for N. In the final refinement a low angle reflection evidently effected by the beam stop was omitted, *i.e*. (1 1 0).

### Crystal data

**Table:**

C_14_H_14_N_4_S_2_·C_14_H_10_O_4_S_2_	*F*(000) = 1264
*M**_r_* = 608.75	*D*_x_ = 1.459 Mg m^−^^3^
Monoclinic, *P*2_1_/*c*	Mo *K*α radiation, λ = 0.71069 Å
Hall symbol: -P 2ybc	Cell parameters from 11634 reflections
*a* = 18.502 (5) Å	θ = 2.2–40.8°
*b* = 10.624 (3) Å	µ = 0.39 mm^−^^1^
*c* = 15.026 (4) Å	*T* = 98 K
β = 110.235 (5)°	Block, red
*V* = 2771.3 (13) Å^3^	0.38 × 0.26 × 0.10 mm
*Z* = 4	

### Data collection

**Table:**

Rigaku AFC12K/SATURN724 diffractometer	5349 reflections with *I* > 2σ(*I*)
Radiation source: fine-focus sealed tube	*R*_int_ = 0.050
graphite	θ_max_ = 27.5°, θ_min_ = 2.3°
ω scans	*h* = −22→24
16758 measured reflections	*k* = −13→12
6348 independent reflections	*l* = −19→12

### Refinement

**Table:**

Refinement on *F*^2^	Primary atom site location: structure-invariant direct methods
Least-squares matrix: full	Secondary atom site location: difference Fourier map
*R*[*F*^2^ > 2σ(*F*^2^)] = 0.063	Hydrogen site location: inferred from neighbouring sites
*wR*(*F*^2^) = 0.156	H atoms treated by a mixture of independent and constrained refinement
*S* = 1.13	*w* = 1/[σ^2^(*F*_o_^2^) + (0.0595*P*)^2^ + 1.5417*P*] where *P* = (*F*_o_^2^ + 2*F*_c_^2^)/3
6348 reflections	(Δ/σ)_max_ = 0.001
373 parameters	Δρ_max_ = 0.40 e Å^−^^3^
4 restraints	Δρ_min_ = −0.30 e Å^−^^3^

### Special details

**Table:**

Geometry. All s.u.'s (except the s.u. in the dihedral angle between two l.s. planes) are estimated using the full covariance matrix. The cell s.u.'s are taken into account individually in the estimation of s.u.'s in distances, angles and torsion angles; correlations between s.u.'s in cell parameters are only used when they are defined by crystal symmetry. An approximate (isotropic) treatment of cell s.u.'s is used for estimating s.u.'s involving l.s. planes.
Refinement. Refinement of *F*^2^ against ALL reflections. The weighted *R*-factor *wR* and goodness of fit *S* are based on *F*^2^, conventional *R*-factors *R* are based on *F*, with *F* set to zero for negative *F*^2^. The threshold expression of *F*^2^ > 2σ(*F*^2^) is used only for calculating *R*-factors(gt) *etc*. and is not relevant to the choice of reflections for refinement. *R*-factors based on *F*^2^ are statistically about twice as large as those based on *F*, and *R*- factors based on ALL data will be even larger.

### Fractional atomic coordinates and isotropic or equivalent isotropic displacement parameters (Å^2^)

**Table:**

	*x*	*y*	*z*	*U*_iso_*/*U*_eq_	
S1	0.14789 (4)	1.09050 (7)	0.26162 (6)	0.0419 (2)	
S2	0.38384 (5)	1.05034 (10)	0.43964 (7)	0.0577 (3)	
S3	0.28281 (4)	0.41538 (6)	0.47637 (5)	0.03595 (18)	
S4	0.23818 (4)	0.28425 (6)	0.54351 (5)	0.03659 (18)	
O1	0.36687 (12)	0.57687 (19)	0.40613 (16)	0.0439 (5)	
O2	0.45371 (13)	0.7063 (3)	0.50168 (18)	0.0542 (6)	
H1o	0.469 (2)	0.707 (4)	0.455 (2)	0.081*	
O3	0.18572 (13)	0.1116 (2)	0.63693 (18)	0.0487 (5)	
O4	0.06256 (14)	0.06603 (19)	0.61426 (18)	0.0482 (6)	
H2o	0.0915 (19)	0.016 (3)	0.655 (2)	0.072*	
N1	0.10469 (15)	0.6043 (2)	0.23918 (19)	0.0389 (6)	
N2	0.22644 (15)	0.9846 (3)	0.42551 (19)	0.0445 (6)	
H1n	0.2727 (10)	0.978 (3)	0.4689 (19)	0.053*	
N3	0.30049 (14)	1.2123 (2)	0.3103 (2)	0.0401 (6)	
H2n	0.2547 (10)	1.233 (3)	0.271 (2)	0.048*	
N4	0.48896 (14)	1.2348 (3)	0.12994 (19)	0.0439 (6)	
C1	0.13990 (17)	0.8058 (3)	0.3642 (2)	0.0363 (6)	
C2	0.19777 (17)	0.7309 (3)	0.3524 (2)	0.0389 (6)	
H2	0.2504	0.7483	0.3872	0.047*	
C3	0.17793 (17)	0.6311 (3)	0.2896 (2)	0.0376 (6)	
H3	0.2176	0.5801	0.2820	0.045*	
C4	0.04915 (17)	0.6752 (3)	0.2516 (2)	0.0389 (6)	
H4	−0.0030	0.6554	0.2163	0.047*	
C5	0.06427 (16)	0.7753 (3)	0.3131 (2)	0.0367 (6)	
H5	0.0233	0.8230	0.3205	0.044*	
C6	0.16015 (19)	0.9164 (3)	0.4317 (2)	0.0465 (7)	
H6A	0.1155	0.9740	0.4164	0.056*	
H6B	0.1717	0.8858	0.4974	0.056*	
C7	0.22516 (16)	1.0588 (3)	0.3541 (2)	0.0365 (6)	
C8	0.30383 (17)	1.1141 (3)	0.3643 (2)	0.0395 (7)	
C9	0.36528 (19)	1.2963 (3)	0.3191 (3)	0.0514 (9)	
H9A	0.4025	1.2903	0.3847	0.062*	
H9B	0.3459	1.3839	0.3093	0.062*	
C10	0.40742 (17)	1.2706 (3)	0.2513 (2)	0.0414 (7)	
C11	0.43798 (18)	1.3709 (3)	0.2185 (3)	0.0476 (7)	
H11	0.4306	1.4543	0.2365	0.057*	
C12	0.47942 (19)	1.3496 (3)	0.1591 (3)	0.0509 (8)	
H12	0.5018	1.4192	0.1385	0.061*	
C13	0.45899 (17)	1.1392 (3)	0.1596 (2)	0.0447 (7)	
H13	0.4657	1.0572	0.1384	0.054*	
C14	0.41768 (18)	1.1519 (3)	0.2208 (3)	0.0470 (7)	
H14	0.3969	1.0802	0.2411	0.056*	
C15	0.35166 (15)	0.6427 (2)	0.5491 (2)	0.0336 (6)	
C16	0.29850 (16)	0.5498 (2)	0.5523 (2)	0.0337 (6)	
C17	0.25988 (17)	0.5629 (3)	0.6169 (2)	0.0384 (6)	
H17	0.2235	0.5011	0.6193	0.046*	
C18	0.27440 (19)	0.6650 (3)	0.6771 (2)	0.0460 (7)	
H18	0.2473	0.6728	0.7203	0.055*	
C19	0.32744 (19)	0.7565 (3)	0.6763 (3)	0.0482 (8)	
H19	0.3373	0.8259	0.7187	0.058*	
C20	0.36576 (17)	0.7446 (3)	0.6123 (2)	0.0407 (7)	
H20	0.4024	0.8067	0.6112	0.049*	
C21	0.39214 (16)	0.6382 (3)	0.4790 (2)	0.0352 (6)	
C22	0.13608 (16)	0.3115 (3)	0.4988 (2)	0.0346 (6)	
C23	0.08651 (17)	0.2328 (2)	0.5271 (2)	0.0351 (6)	
C24	0.00720 (18)	0.2556 (3)	0.4900 (2)	0.0418 (7)	
H24	−0.0266	0.2019	0.5078	0.050*	
C25	−0.02326 (18)	0.3530 (3)	0.4287 (2)	0.0450 (7)	
H25	−0.0773	0.3669	0.4047	0.054*	
C26	0.02518 (19)	0.4301 (3)	0.4026 (2)	0.0466 (7)	
H26	0.0046	0.4987	0.3610	0.056*	
C27	0.10406 (17)	0.4090 (3)	0.4363 (2)	0.0415 (7)	
H27	0.1367	0.4622	0.4161	0.050*	
C28	0.11699 (17)	0.1310 (3)	0.5979 (2)	0.0369 (6)	

### Atomic displacement parameters (Å^2^)

**Table:**

	*U*^11^	*U*^22^	*U*^33^	*U*^12^	*U*^13^	*U*^23^
S1	0.0356 (4)	0.0497 (4)	0.0434 (5)	−0.0029 (3)	0.0175 (3)	0.0003 (3)
S2	0.0405 (4)	0.0839 (6)	0.0467 (5)	−0.0025 (4)	0.0127 (4)	−0.0042 (5)
S3	0.0454 (4)	0.0380 (4)	0.0333 (4)	−0.0046 (3)	0.0250 (3)	−0.0045 (3)
S4	0.0435 (4)	0.0368 (3)	0.0357 (4)	−0.0001 (3)	0.0215 (3)	0.0020 (3)
O1	0.0499 (12)	0.0498 (12)	0.0446 (13)	−0.0110 (9)	0.0321 (11)	−0.0096 (10)
O2	0.0440 (12)	0.0843 (16)	0.0417 (14)	−0.0211 (11)	0.0244 (11)	−0.0103 (13)
O3	0.0585 (14)	0.0433 (11)	0.0520 (14)	0.0064 (10)	0.0292 (12)	0.0153 (10)
O4	0.0566 (14)	0.0402 (11)	0.0561 (15)	−0.0004 (10)	0.0300 (12)	0.0117 (10)
N1	0.0505 (14)	0.0356 (12)	0.0407 (14)	−0.0035 (10)	0.0285 (12)	−0.0009 (10)
N2	0.0473 (14)	0.0528 (15)	0.0324 (14)	−0.0135 (12)	0.0125 (12)	−0.0055 (12)
N3	0.0408 (13)	0.0452 (13)	0.0427 (15)	−0.0110 (11)	0.0253 (12)	−0.0135 (11)
N4	0.0355 (12)	0.0631 (16)	0.0361 (15)	0.0073 (11)	0.0160 (11)	0.0018 (12)
C1	0.0486 (16)	0.0363 (13)	0.0296 (15)	−0.0040 (12)	0.0205 (13)	0.0023 (11)
C2	0.0376 (14)	0.0440 (15)	0.0368 (17)	0.0005 (12)	0.0152 (13)	0.0100 (12)
C3	0.0442 (15)	0.0341 (13)	0.0431 (17)	0.0045 (11)	0.0262 (14)	0.0100 (12)
C4	0.0417 (15)	0.0393 (14)	0.0399 (17)	−0.0047 (12)	0.0197 (13)	−0.0018 (12)
C5	0.0422 (15)	0.0372 (13)	0.0379 (16)	−0.0005 (11)	0.0231 (13)	−0.0007 (12)
C6	0.0573 (19)	0.0523 (17)	0.0356 (17)	−0.0154 (15)	0.0231 (15)	−0.0088 (14)
C7	0.0423 (15)	0.0377 (14)	0.0346 (16)	−0.0074 (11)	0.0200 (13)	−0.0092 (12)
C8	0.0415 (15)	0.0451 (15)	0.0371 (16)	−0.0088 (12)	0.0202 (13)	−0.0160 (13)
C9	0.0545 (19)	0.0558 (18)	0.058 (2)	−0.0189 (15)	0.0367 (18)	−0.0220 (16)
C10	0.0378 (15)	0.0480 (16)	0.0438 (18)	−0.0066 (12)	0.0210 (14)	−0.0065 (13)
C11	0.0480 (17)	0.0491 (17)	0.051 (2)	0.0005 (14)	0.0241 (16)	−0.0003 (15)
C12	0.0492 (18)	0.0589 (19)	0.052 (2)	0.0012 (15)	0.0273 (17)	0.0020 (16)
C13	0.0417 (16)	0.0509 (17)	0.0427 (18)	0.0026 (13)	0.0160 (14)	−0.0075 (14)
C14	0.0492 (17)	0.0469 (16)	0.051 (2)	−0.0080 (13)	0.0248 (16)	−0.0093 (14)
C15	0.0333 (13)	0.0394 (14)	0.0313 (15)	−0.0002 (11)	0.0154 (12)	−0.0004 (11)
C16	0.0378 (14)	0.0370 (13)	0.0300 (15)	0.0011 (11)	0.0165 (12)	−0.0008 (11)
C17	0.0444 (15)	0.0408 (14)	0.0382 (17)	−0.0022 (12)	0.0246 (14)	−0.0037 (12)
C18	0.0568 (18)	0.0523 (17)	0.0413 (18)	−0.0017 (14)	0.0326 (16)	−0.0079 (14)
C19	0.0574 (19)	0.0482 (17)	0.0459 (19)	−0.0053 (14)	0.0267 (16)	−0.0164 (14)
C20	0.0425 (15)	0.0432 (15)	0.0402 (17)	−0.0062 (12)	0.0190 (14)	−0.0060 (13)
C21	0.0363 (14)	0.0389 (14)	0.0347 (16)	0.0012 (11)	0.0178 (12)	0.0008 (12)
C22	0.0418 (14)	0.0375 (13)	0.0291 (15)	−0.0049 (11)	0.0182 (12)	−0.0040 (11)
C23	0.0470 (15)	0.0350 (13)	0.0279 (14)	−0.0040 (11)	0.0187 (12)	−0.0023 (11)
C24	0.0481 (16)	0.0431 (15)	0.0391 (17)	−0.0109 (13)	0.0213 (14)	−0.0024 (13)
C25	0.0416 (16)	0.0552 (18)	0.0354 (17)	−0.0042 (13)	0.0099 (14)	0.0021 (14)
C26	0.0497 (17)	0.0499 (17)	0.0370 (18)	−0.0036 (14)	0.0108 (14)	0.0088 (13)
C27	0.0449 (16)	0.0456 (16)	0.0359 (17)	−0.0068 (13)	0.0166 (14)	0.0065 (13)
C28	0.0494 (17)	0.0323 (13)	0.0373 (16)	−0.0010 (12)	0.0255 (14)	−0.0020 (11)

### Geometric parameters (Å, °)

**Table:**

S1—C7	1.648 (3)	C9—C10	1.506 (4)
S2—C8	1.665 (3)	C9—H9A	0.9900
S3—C16	1.788 (3)	C9—H9B	0.9900
S3—S4	2.0535 (10)	C10—C11	1.375 (4)
S4—C22	1.796 (3)	C10—C14	1.378 (4)
O1—C21	1.219 (4)	C11—C12	1.381 (4)
O2—C21	1.292 (3)	C11—H11	0.9500
O2—H1o	0.84 (3)	C12—H12	0.9500
O3—C28	1.220 (4)	C13—C14	1.390 (4)
O4—C28	1.312 (3)	C13—H13	0.9500
O4—H2o	0.85 (3)	C14—H14	0.9500
N1—C3	1.334 (4)	C15—C20	1.404 (4)
N1—C4	1.338 (4)	C15—C16	1.406 (4)
N2—C7	1.325 (4)	C15—C21	1.490 (4)
N2—C6	1.455 (4)	C16—C17	1.397 (4)
N2—H1n	0.88 (3)	C17—C18	1.379 (4)
N3—C8	1.310 (4)	C17—H17	0.9500
N3—C9	1.464 (4)	C18—C19	1.384 (4)
N3—H2n	0.88 (3)	C18—H18	0.9500
N4—C13	1.308 (4)	C19—C20	1.384 (4)
N4—C12	1.329 (4)	C19—H19	0.9500
C1—C5	1.382 (4)	C20—H20	0.9500
C1—C2	1.394 (4)	C22—C27	1.386 (4)
C1—C6	1.512 (4)	C22—C23	1.411 (4)
C2—C3	1.382 (4)	C23—C24	1.399 (4)
C2—H2	0.9500	C23—C28	1.485 (4)
C3—H3	0.9500	C24—C25	1.371 (4)
C4—C5	1.373 (4)	C24—H24	0.9500
C4—H4	0.9500	C25—C26	1.367 (4)
C5—H5	0.9500	C25—H25	0.9500
C6—H6A	0.9900	C26—C27	1.388 (4)
C6—H6B	0.9900	C26—H26	0.9500
C7—C8	1.527 (4)	C27—H27	0.9500
			
C16—S3—S4	103.44 (10)	N4—C12—C11	122.0 (3)
C22—S4—S3	104.86 (10)	N4—C12—H12	119.0
C21—O2—H1O	108 (3)	C11—C12—H12	119.0
C28—O4—H2O	98 (3)	N4—C13—C14	123.0 (3)
C3—N1—C4	118.6 (3)	N4—C13—H13	118.5
C7—N2—C6	124.7 (3)	C14—C13—H13	118.5
C7—N2—H1N	113 (2)	C10—C14—C13	118.7 (3)
C6—N2—H1N	122 (2)	C10—C14—H14	120.6
C8—N3—C9	124.6 (3)	C13—C14—H14	120.6
C8—N3—H2N	117 (2)	C20—C15—C16	119.1 (3)
C9—N3—H2N	118 (2)	C20—C15—C21	118.8 (2)
C13—N4—C12	118.7 (3)	C16—C15—C21	122.0 (2)
C5—C1—C2	118.1 (3)	C17—C16—C15	119.1 (3)
C5—C1—C6	121.5 (3)	C17—C16—S3	120.9 (2)
C2—C1—C6	120.4 (3)	C15—C16—S3	120.0 (2)
C3—C2—C1	119.4 (3)	C18—C17—C16	120.3 (3)
C3—C2—H2	120.3	C18—C17—H17	119.9
C1—C2—H2	120.3	C16—C17—H17	119.9
N1—C3—C2	121.9 (3)	C17—C18—C19	121.6 (3)
N1—C3—H3	119.0	C17—C18—H18	119.2
C2—C3—H3	119.0	C19—C18—H18	119.2
N1—C4—C5	122.9 (3)	C20—C19—C18	118.5 (3)
N1—C4—H4	118.6	C20—C19—H19	120.7
C5—C4—H4	118.6	C18—C19—H19	120.7
C4—C5—C1	119.1 (3)	C19—C20—C15	121.4 (3)
C4—C5—H5	120.4	C19—C20—H20	119.3
C1—C5—H5	120.4	C15—C20—H20	119.3
N2—C6—C1	111.3 (2)	O1—C21—O2	124.4 (3)
N2—C6—H6A	109.4	O1—C21—C15	121.5 (3)
C1—C6—H6A	109.4	O2—C21—C15	114.1 (3)
N2—C6—H6B	109.4	C27—C22—C23	118.5 (3)
C1—C6—H6B	109.4	C27—C22—S4	121.5 (2)
H6A—C6—H6B	108.0	C23—C22—S4	120.1 (2)
N2—C7—C8	113.5 (3)	C24—C23—C22	118.6 (3)
N2—C7—S1	124.9 (2)	C24—C23—C28	119.9 (2)
C8—C7—S1	121.6 (2)	C22—C23—C28	121.5 (3)
N3—C8—C7	113.9 (3)	C25—C24—C23	122.0 (3)
N3—C8—S2	125.8 (2)	C25—C24—H24	119.0
C7—C8—S2	120.3 (2)	C23—C24—H24	119.0
N3—C9—C10	115.3 (3)	C26—C25—C24	119.1 (3)
N3—C9—H9A	108.4	C26—C25—H25	120.4
C10—C9—H9A	108.4	C24—C25—H25	120.4
N3—C9—H9B	108.4	C25—C26—C27	120.7 (3)
C10—C9—H9B	108.4	C25—C26—H26	119.7
H9A—C9—H9B	107.5	C27—C26—H26	119.7
C11—C10—C14	117.9 (3)	C22—C27—C26	121.1 (3)
C11—C10—C9	118.4 (3)	C22—C27—H27	119.4
C14—C10—C9	123.7 (3)	C26—C27—H27	119.4
C10—C11—C12	119.6 (3)	O3—C28—O4	124.2 (3)
C10—C11—H11	120.2	O3—C28—C23	122.8 (3)
C12—C11—H11	120.2	O4—C28—C23	113.0 (3)
			
C16—S3—S4—C22	88.06 (13)	C20—C15—C16—S3	−177.1 (2)
C5—C1—C2—C3	1.1 (4)	C21—C15—C16—S3	4.5 (4)
C6—C1—C2—C3	−179.4 (3)	S4—S3—C16—C17	−20.3 (3)
C4—N1—C3—C2	−1.3 (4)	S4—S3—C16—C15	158.2 (2)
C1—C2—C3—N1	0.3 (4)	C15—C16—C17—C18	−0.6 (4)
C3—N1—C4—C5	0.8 (4)	S3—C16—C17—C18	177.9 (2)
N1—C4—C5—C1	0.6 (4)	C16—C17—C18—C19	−0.5 (5)
C2—C1—C5—C4	−1.5 (4)	C17—C18—C19—C20	0.7 (5)
C6—C1—C5—C4	178.9 (3)	C18—C19—C20—C15	0.1 (5)
C7—N2—C6—C1	73.4 (4)	C16—C15—C20—C19	−1.2 (5)
C5—C1—C6—N2	−139.7 (3)	C21—C15—C20—C19	177.3 (3)
C2—C1—C6—N2	40.8 (4)	C20—C15—C21—O1	−157.7 (3)
C6—N2—C7—C8	−177.5 (3)	C16—C15—C21—O1	20.7 (4)
C6—N2—C7—S1	2.1 (4)	C20—C15—C21—O2	20.7 (4)
C9—N3—C8—C7	168.0 (2)	C16—C15—C21—O2	−160.9 (3)
C9—N3—C8—S2	−11.6 (4)	S3—S4—C22—C27	−3.0 (3)
N2—C7—C8—N3	−162.1 (3)	S3—S4—C22—C23	177.0 (2)
S1—C7—C8—N3	18.3 (3)	C27—C22—C23—C24	0.8 (4)
N2—C7—C8—S2	17.4 (3)	S4—C22—C23—C24	−179.2 (2)
S1—C7—C8—S2	−162.20 (17)	C27—C22—C23—C28	−176.5 (3)
C8—N3—C9—C10	98.3 (4)	S4—C22—C23—C28	3.5 (4)
N3—C9—C10—C11	146.4 (3)	C22—C23—C24—C25	−1.2 (4)
N3—C9—C10—C14	−34.1 (5)	C28—C23—C24—C25	176.1 (3)
C14—C10—C11—C12	−2.0 (5)	C23—C24—C25—C26	0.3 (5)
C9—C10—C11—C12	177.6 (3)	C24—C25—C26—C27	1.1 (5)
C13—N4—C12—C11	−1.0 (5)	C23—C22—C27—C26	0.6 (5)
C10—C11—C12—N4	2.1 (5)	S4—C22—C27—C26	−179.5 (3)
C12—N4—C13—C14	−0.2 (5)	C25—C26—C27—C22	−1.5 (5)
C11—C10—C14—C13	0.8 (5)	C24—C23—C28—O3	−175.5 (3)
C9—C10—C14—C13	−178.7 (3)	C22—C23—C28—O3	1.7 (4)
N4—C13—C14—C10	0.3 (5)	C24—C23—C28—O4	3.9 (4)
C20—C15—C16—C17	1.4 (4)	C22—C23—C28—O4	−178.8 (3)
C21—C15—C16—C17	−177.0 (3)		

### Hydrogen-bond geometry (Å, °)

**Table:**

*D*—H···*A*	*D*—H	H···*A*	*D*···*A*	*D*—H···*A*
O2—H1o···N4^i^	0.84 (3)	1.73 (3)	2.565 (4)	168 (4)
O4—H2o···N1^ii^	0.85 (3)	1.76 (3)	2.529 (3)	151 (4)
N2—H1n···S2	0.88 (2)	2.37 (2)	2.930 (3)	121 (2)
N3—H2n···O3^iii^	0.88 (3)	2.58 (3)	3.312 (4)	142 (2)
N3—H2n···S1	0.88 (3)	2.45 (3)	2.959 (3)	117 (2)

Symmetry codes: (i) −*x*+1, *y*−1/2, −*z*+1/2; (ii) *x*, −*y*+1/2, *z*+1/2; (iii) *x*, −*y*+3/2, *z*−1/2.
